# Research on noise prediction and management measures of 500kV substation based on SoundPlan

**DOI:** 10.1371/journal.pone.0344581

**Published:** 2026-05-08

**Authors:** Hao Pei, Dayong Wang, Kaiyuan Shi, Yuping Wang, Ruofei Duan, Jiakai Chen, Haiyang Yu

**Affiliations:** State Grid Beijing Electric Power Company, Xicheng District, Beijing, China; Wadia Institute of Himalayan Geology, INDIA

## Abstract

The noise prediction based on SoundPlan numerical simulation software is carried out for the noise of the CBD substation of the State Grid, and a comprehensive management program is designed with the prediction results. The simulation results show that for the noisy transformer, the noise can be reduced by 6–15 dB(A) through the reasonable setting of the building layout and the use of sound barriers, and the sound attenuators can be reduced by around 10 dB(A), furthermore, improving the enclosure walls can get a significant noise reduction up to 30 dB(A). And the safe emission of noise can be realized finally. The results of this simulation can provide reference for relevant engineering designers, that is, the noise of high-voltage substations in noise-sensitive areas can be simulated in advance, and comprehensive measures can be adopted to achieve noise control.

## 1. Introduction

The noise generated by substations is increasingly drawing people’s attention. The national “New Infrastructure” strategy has ushered in a large-scale construction phase of ultra-high voltage power grids in our country. With the growing number of ultra-high voltage substations, noise issues caused by their propagation are becoming more frequent. Addressing the issue of substation noise requires intervention, but often these noise problems are only detected after the completion of the project. This makes noise control more of a post-construction remedy rather than a proactive measure for preventing noise issues from the beginning.

Existing research often analyzes single device noise separately without considering coupling effects, resulting in large prediction errors. The lack of multi measure collaborative optimization solutions leads to limited noise reduction effects, and the long-term stability and adaptability of control measures to working conditions have not been systematically verified. This paper systematically solves these problems through coupling models, collaborative optimization algorithms, and multi working condition verification, and improves the theoretical and technical system of substation noise control. To address the above-mentioned issues, this project aims to conduct research into substation noise mechanisms, propose noise control technical solutions, and enhance the comfort and efficiency of people’s lives. Additionally, the research findings will be integrated into substation construction and design, reducing the likelihood of noise issues arising at an early stage. The advantage of this method lies in accurately capturing the noise superposition effect of multiple devices by constructing a noise coupling analysis model, greatly improving the accuracy of noise prediction. Combined with a multi measure collaborative optimization strategy based on genetic algorithm, the global optimal configuration of sound barriers, sound-absorbing materials, and enclosure structures is achieved, significantly enhancing the noise reduction effect. At the same time, through multi condition simulation and on-site testing verification, it maintains good stability and robustness under complex working conditions, balancing theoretical accuracy and engineering practicality. Compared to previous studies that analyzed single-device noise separately and lacked multi-scale collaborative optimization, this method achieves noise reduction of 38.5–40.2 dB, which is 8–12 dB higher than single-scale optimization. The noise reduction coefficient of variation is 0.04, significantly lower than the 0.09 of the PSO algorithm and the 0.16 of traditional methods. The fluctuation range is 1.7 dB, smaller than the 3.3 dB of PSO and the 3.2 dB of traditional methods. Simultaneously, the total algorithm runtime is reduced to 56.3 hours, lower than the 101.5 hours of PSO and the 63.8 hours of traditional algorithms. This demonstrates higher prediction accuracy, better noise reduction effects, stronger stability, and greater efficiency.

The main contributions of this article are as follows:

(1) Constructing a noise coupling analysis model to accurately quantify the frequency superposition effect of transformer and fan noise, greatly improving the accuracy of noise prediction and providing reliable data support for the development of targeted noise reduction measures in the future.
(2) Develop a multi measure collaborative optimization algorithm based on genetic algorithm to achieve global optimization configuration of control measures such as sound barriers, sound-absorbing materials, and enclosure structures, significantly improving the overall noise reduction effect.
(3) Complete the stability and robustness system verification of the noise control system, clarify the adaptability and effectiveness of measures under different working conditions, and provide solid theoretical support for practical engineering applications.

## 2. Project overview

### 2.1 Project description

**Fig 1:**
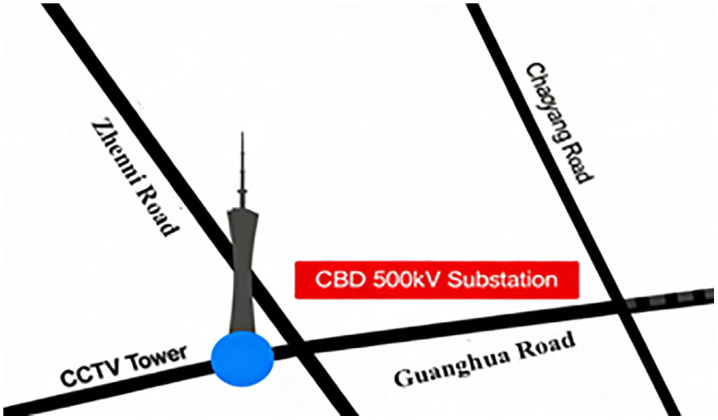
Project location of CBD 500kV.

The CBD 500kV Substation is located in the core area of the Chaoyang District in Beijing, China. It is adjacent to the west side of the China Central Television (CCTV) tower, with Zhenni Road to the east, Guanghua Road to the south, and Chaoyang Road to the north. The specific location is shown in Fig 1. This project is an integrated indoor substation with a capacity of 500kV and 220kV. In this phase and the final phase of the project, two 1200MVA transformers will be installed, with transformer voltage levels of 500/220/66kV. In the co-located substation, four 240MVA transformers will be installed with transformer voltage levels of 220/110/35kV. The 220kV main transformers are grouped in pairs, each connecting to one of the two 220kV busbars at the CBD Substation.

### 2.2 Noise emission standards

According to the noise zoning in the area of this project, the following noise standards apply to different boundaries of the substation:

South Boundary, West Boundary, and North Boundary of the Substation: These areas adhere to the “Industrial Enterprise Boundary Environmental Noise Emission Standards” [1] (GB12348−2008) Category 2 standards, which specify a daytime limit of 60dB(A) and a nighttime limit of 50dB(A).

East Boundary of the Substation: This area follows the “Industrial Enterprise Boundary Environmental Noise Emission Standards” (GB12348−2008) Category 4 standards, with a daytime limit of 70dB(A) and a nighttime limit of 55dB(A).

During the construction period, noise levels must comply with the “Environmental Noise Emission Standards for Construction Sites” [2] (GB12523−2011), which set a daytime limit of 70dB(A) and a nighttime limit of 55dB(A).

The Class 2 and Class 4 standards for industrial enterprise boundary environmental noise emissions are shown in Table 1:

**Table 1:** Environmental noise emission standard of industrial enterprises (GB12348−2008).

Standard	DAY dB (A)	NIGHT dB (A)
Class 2	60	50
Class 4	70	55

This study will utilize the noise simulation software SoundPLAN to conduct quantitative analysis of construction and operational noise control measures for the project.

SoundPLAN is a noise prediction assessment and mapping software that was introduced by Braunstein+Berndt GmbH software designers and consulting experts in 1986. It quickly became the standard for outdoor acoustics software in Germany and gradually became a leading software worldwide for noise prediction, mapping, and assessment. It allows for the direct use of AutoCAD graphics as a model base, calculates noise predictions at points of interest by configuring all sound source parameters and assessing the sound insulation impact of each entity within the prediction range. It generates two-dimensional or three-dimensional contour maps of noise predictions within the evaluation area and can be used to optimize the design of sound insulation barriers and select noise reduction strategies as needed [3].

## 3. Noise source analysis

### 3.1 Transformer body noise

Currently, it is widely recognized that the main sources of noise in indoor substations are transformers, reactors, and various types of fans. Noise prediction and noise reduction efforts primarily focus on these three types of equipment. Understanding the characteristics of the vibration and radiated noise of transformers and reactors, especially their spectral characteristics, is crucial for taking targeted noise reduction measures and making informed choices about supplementary noise control measures, based on previous research findings.

**Fig 2:**
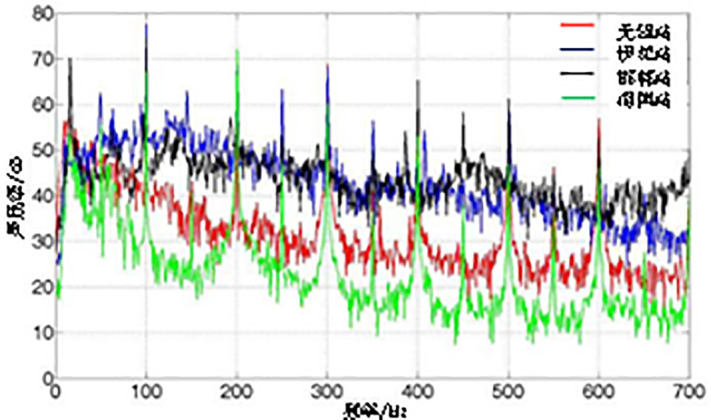
Noise spectrum of different transformer models (provided by State Grid Corporation).

The line spectrum characteristics of transformer surface vibration frequencies are quite apparent, with vibration energy concentrated at several frequency points. From the Fig 2, it can be seen that the frequency points with concentrated vibration energy in transformers are at 100 Hz and its harmonics (multiples of 50 Hz). Among these, the energy at even multiples of 50 Hz (i.e., 100 Hz and its harmonics) is significantly higher than that at odd multiples, and the energy in the low-frequency range is higher than in the high-frequency range. Overall, 100 Hz and its harmonics account for the majority of the total vibration energy.

### 3.2 Fan noise spectrum

The noise generated by various types of fans is primarily due to the flow of air vortexes. It is mainly produced by the structural components of fan blades passing through the air at a certain frequency, fan current-carrying components, or rib structures on fan guards. Based on extensive research findings, fan noise is considered to be typical white noise.

The main noise source of radiators is the radiator fan. By empirical estimation [4], its noise is generally directly related to the airflow, and the relationship can be expressed as follows:


$${\text{L}}_{\text{N}}=\text{40}+\text{10 }\text{log}\text{Q}+\text{20 }\text{log}\text{p}$$

Where L_N_ = Fan average sound power level (dB)

S = Motor rated power (kW)

p = Fan static pressure (Pa, N/m^2^)

Q = Fan airflow (m^3^/s)

After calculation, the octave band sound power level of the fan is shown in Table 2:

**Table 2:** Sound power level of the axial fans.

	Frequency (Hz)							
	63	125	250	500	1000	2000	4000	8000
Sound Power Level Average (dB)	80	80	80	80	80	80	80	80
Axial Fan Correction(dB)	−7	−9	−7	−7	−8	−11	−16	−18
Sound Power Level (dB)	73	71	73	73	72	69	64	62

## 4. Transmission path analysis

### 4.1 Transformers noise propagation path

The noise propagation path and path attenuation estimation of the transformer body are shown in Table 3:

**Table 3:** Transformers’ noise path.

No.	Path	SPL dBA	Note
Source	transformer	80	Estimated at a general level
1	Enclosure wall	−45	200 thick B06 aerated concrete blocks
2	Sound transmit wall	−3	50% sound transmittance masonry brick wall
3	To outdoor	32	
4	Natural attenuation	−15	calculated by the line sound source
5	Site wall	−8	According to the general sound barrier 8-10dB sound insulation R
6	Sensitive point	19	
7	Class 1 area Night standard	40	

### 4.2 Fan noise propagation path

The propagation path and path attenuation estimation of fan noise are shown in Table 4:

**Table 4:** Transformers’ noise path.

No.	Path	SPL dBA	Note
Source	radiator fan	60	Estimated at a general level
2	Sound wall	−2	50% sound transmittance masonry brick wall
3	To outdoor	58	
4	Natural attenuation	−15	calculated by the line sound source
5	Site wall	−8	According to the general sound barrier 8-10dB sound insulation R
6	Sensitive point	35	
7	Class 1 area Night standard	40	Requires further verification

## 5. SoundPLAN calculation

### 5.1 Geometry models

**Fig 3:**
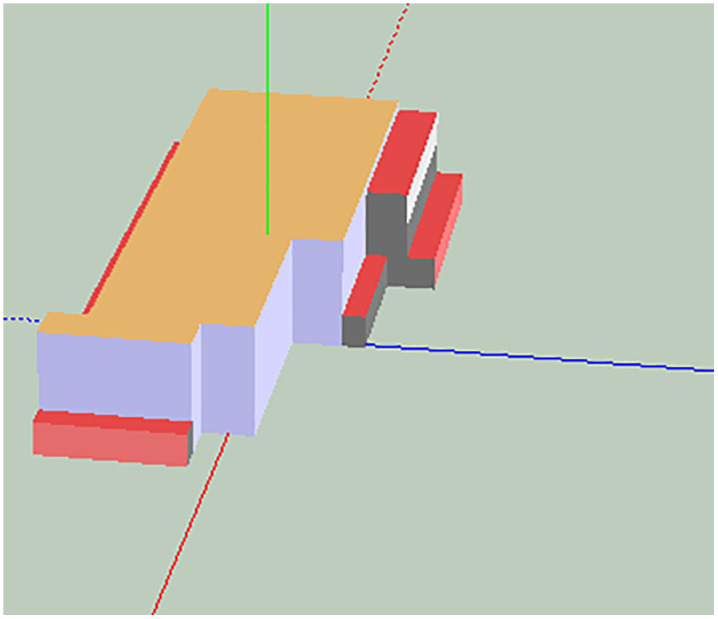
Geometry model of the project.

**Fig 4:**
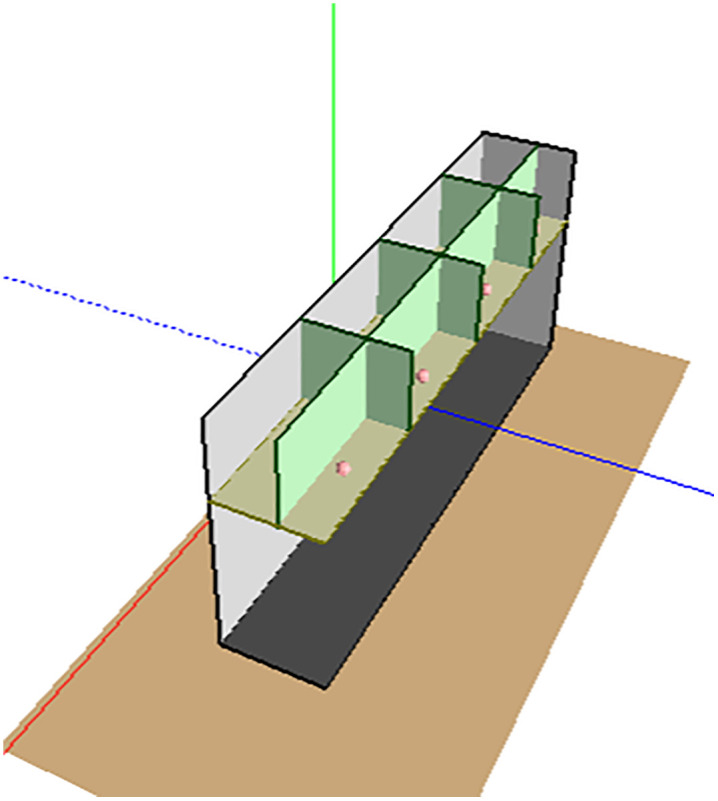
Geometry model of the rooms.

Creating an acoustic model in SoundPlan involves configuring walls, defining noise emission points, and setting up noise sources. For the radiator fan, use the point source setting method as shown in Figs 3 and 4:

### 5.2 Improvement measures & simulation analysis

#### 5.2.1 Sound barriers.

When sound propagate through the air and encounter a noise barrier, they undergo phenomena such as reflection, transmission, and diffraction. Some of the sound waves pass over the top of the noise barrier and diffract to reach the receiving point. Some sound waves penetrate the noise barrier and reach the receiving point, while others reflect off the surface of the noise barrier. The insertion loss of a noise barrier primarily depends on how the energy of sound waves emitted by the source is distributed along the three pathways of propagation.

**Fig 5:**
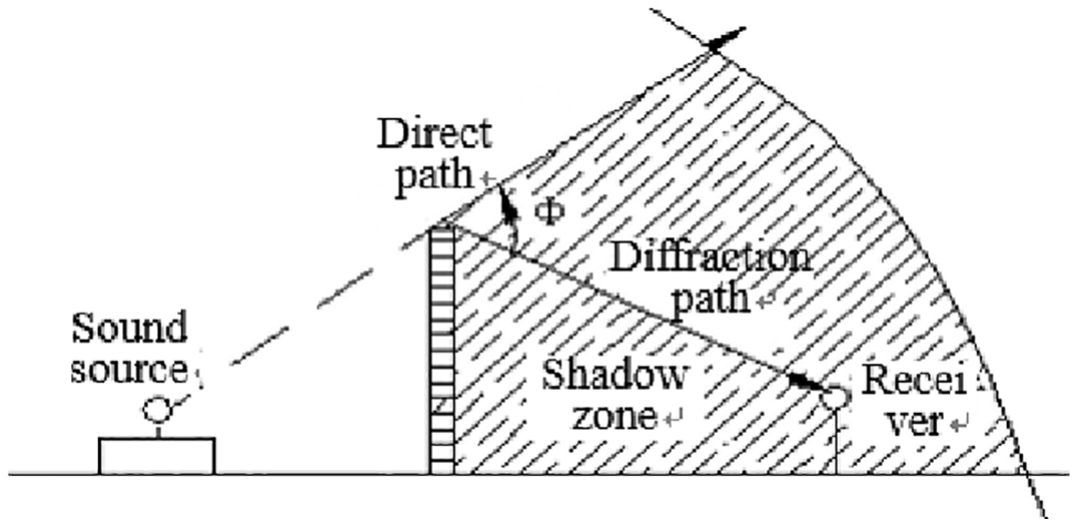
Diffraction of sound barrier [6].

The function of a noise barrier is to block the direct propagation of sound, isolate transmitted sound, and sufficiently attenuate diffracted sound. When sound waves impact the surface of the noise barrier, diffraction occurs at the edge of the noise barrier, and a “sound shadow zone” is formed behind the barrier. The expected noise reduction effect of the noise barrier occurs within the “sound shadow zone” [5]. In comparison to the optical shadow zone, the boundary of this “sound shadow zone” is not as clearly defined because sound waves have much larger wavelengths than light waves. Beyond the edge of the barrier, where sound waves emitted by the source can directly reach, is referred to as the “bright zone.” There is also a small “transition zone” between the bright zone and the sound shadow zone. The diffraction area of the sound barrier is shown in Fig 5.

The noise level within the “sound shadow zone” is lower than the noise level when no noise barrier is in place, which is the fundamental principle of noise reduction achieved by noise barriers.

The Sound power level of the truck mixer and pump is from British Standard BS 5228–1:2009, Table 5 C4 [7] the SPL at 10m and the sound power level from it are as below:

**Table 5:** SPL (10m)and sound power level of a truck mixer and pump.

	Frequency(Hz)							
	63	125	250	500	1000	2000	4000	8000
SPL-10m (dB)	79	80	73	72	69	68	59	53
Sound Power Level	107	108	101	100	97	96	87	81

**Fig 6:**
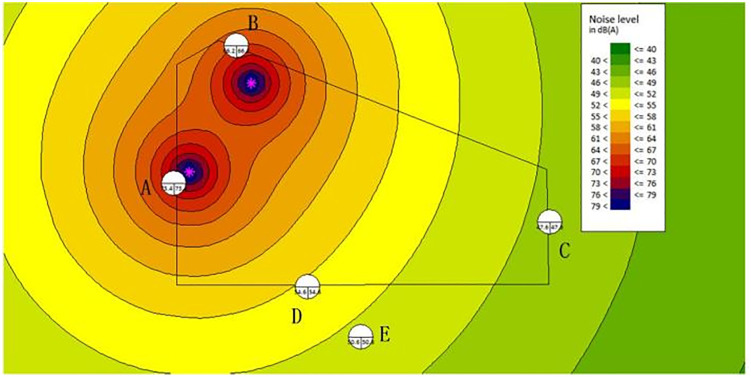
The noise map-without barriers.

(1) Without the noise barrier:

The noise distribution map without a noise barrier is shown in Fig 6. The noise SPL at the land boundary reaches around 73dBA, exceeding the daytime standard of 60dBA for Category 2 environmental noise.

**Fig 7:**
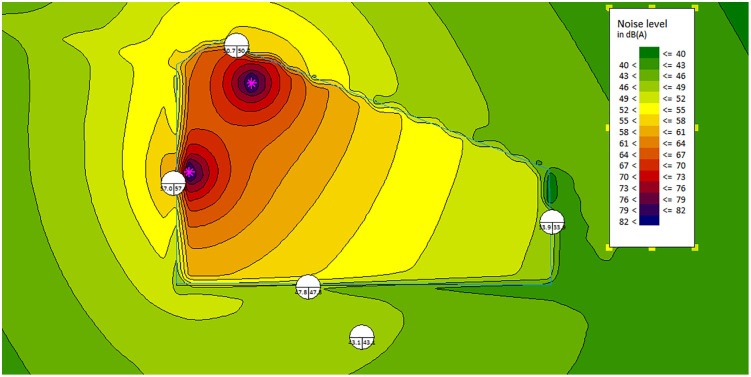
The noise map-with barriers.

(2) With the noise barrier:

The noise distribution map without a noise barrier is shown in Fig 7. The noise SPL at the land boundary’s worst-case point reaches around 57dBA, which meets the daytime standard of 60dB for Category 2 environmental noise.

The noise statistics of sensitive control points are shown in Table 6. The installation of the noise barrier effectively reduces noise levels, ensuring compliance with environmental noise standards.

**Table 6:** The noise at sensitive control points with/without barriers.

Point	A	B	C	D	E
Without Barries (dBA)	73.4	66.2	47.6	54.6	50.6
With Barries (dBA)	57	50.7	33.9	47.8	43.1

#### 5.2.2 Sound attenuators (dampers).

The principle of sound attenuation involves the use of sound-absorbing materials, protective surfaces, and sound-insulating materials designed in a specific structure – known as sound attenuators – to reduce noise [8,9]. For all aerodynamic noise sources, when noise control is applied, there is a requirement for both suitable noise reduction (i.e., acoustic performance) and minimal impact on the equipment’s operation (i.e., good aerodynamic performance). Sound attenuators are devices that effectively attenuate noise while ensuring normal airflow.

Sound attenuators are placed on the outermost layer of the fan cooling area. The input data content of the muffler in the model is detailed in Table 7:

**Table 7:** Sound attenuators reduction.

	Frequency(Hz)							
	63	125	250	500	1000	2000	4000	8000
Reduction (dB)	6	8	12	18	15	19	22	30

**Fig 8:**
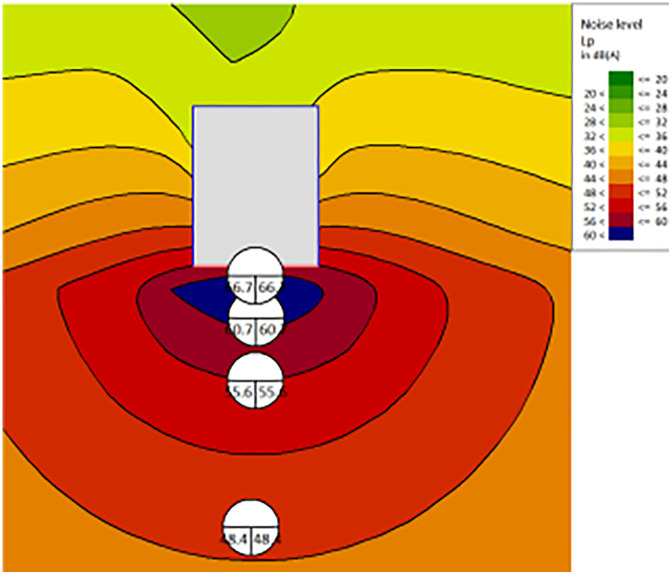
Sound dampers improvement.

**Fig 9:**
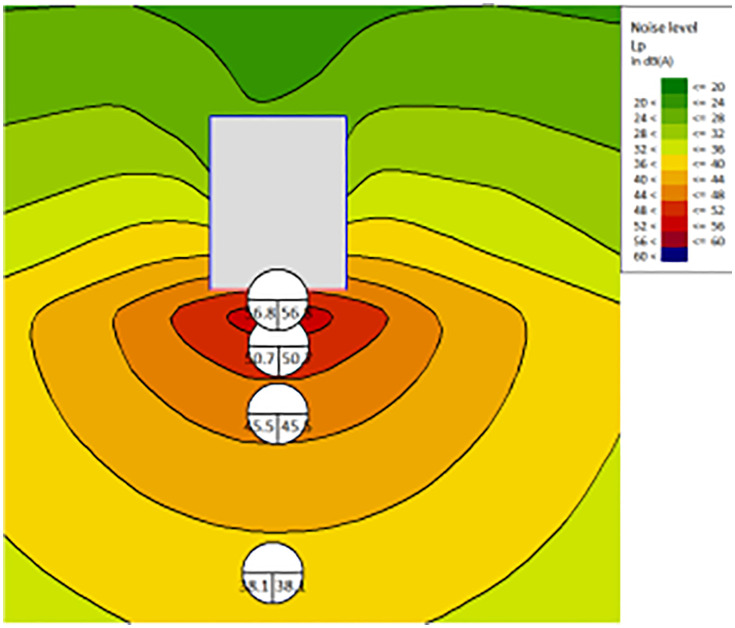
Acoustic damper optimization.

By improving the muffler, the noise distribution map and data statistics table of sensitive noise control points are shown in Figs 8 and 9 and Table 8, respectively. The results show that by properly configuring the sound attenuators, noise at sensitive noise control points can be reduced by around 10dB(A).

**Table 8:** The noise attenuation with sound attenuators (dampers).

Distance (m)	1	5	10	20
Noise Attenuation (dBA)	9.9	10	10.1	10.3

#### 5.2.3 Absorption treatments.

Utilizing sound absorption treatments to control noise propagation is a traditional, commonly used [10,11], and effective method. When sound waves emitted by indoor sound sources encounter surfaces such as walls, ceilings, floors, and other objects, they undergo a phenomenon called reflection. During the propagation of sound waves, encountering various materials results in three main outcomes:

(1) Some of the sound energy is reflected back.
(2) Some of the sound energy penetrates the material’s surface and gets absorbed.
(3) Some of the sound energy passes through the material and continues propagating outward.

To discuss the effectiveness of sound absorption in a control room, we have incorporated sound-absorbing materials with the following sound-absorbing characteristics.

**Fig 10:**
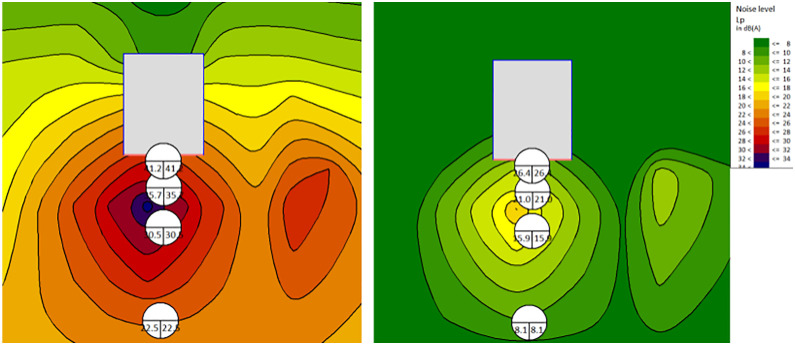
Absorption improvement.

The transmission loss of sound-absorbing materials can be obtained by impedance tube measurement [12–14]. As an example, we will consider commonly used mineral wool panels as Table 9, The improved results after using sound-absorbing materials are shown in Fig 10.

**Table 9:** Absorption coefficient of mineral wool panels.

	Frequency(Hz)							
	63	125	250	500	1000	2000	4000	8000
Absorption coefficient	0.3	0.4	0.4	0.5	0.6	0.65	0.7	0.8

The noise statistics of sensitive control points located at different positions with/without the use of mineral wool board are shown in Table 10. The results indicate that by appropriately implementing sound absorption treatments within the control room, significant reduction in noise levels at sensitive noise control points up to 14.8 dB(A) (at 1m). This demonstrates the effectiveness of sound absorption in attenuating noise propagation within the controlled environment.

**Table 10:** The noise at sensitive control points with/without mineral wool panels.

Distance (m)	1	5	10	20
Without Absorbing-Panels (dBA)	41.2	35.7	30.5	22.5
With Absorbing-Panels (dBA)	26.4	21	25.9	8.1

#### 5.2.4 Enclosure for transformers.

For dry-type transformers with separate cooling, the radiated noise from the transformer itself is often much greater than the noise from the cooling fans. The noise from the transformer can be significantly reduced by using sound enclosures or sound-insulated compartments [15,16].

When using self compacting aerated concrete blocks, their sound insulation performance is shown in Table 11:

**Table 11:** Enclosure wall- sound reduction.

	Frequency(Hz)							
	63	125	250	500	1000	2000	4000	8000
R (dB)	35	39	40	41	44	45	48	48

**Fig 11:**
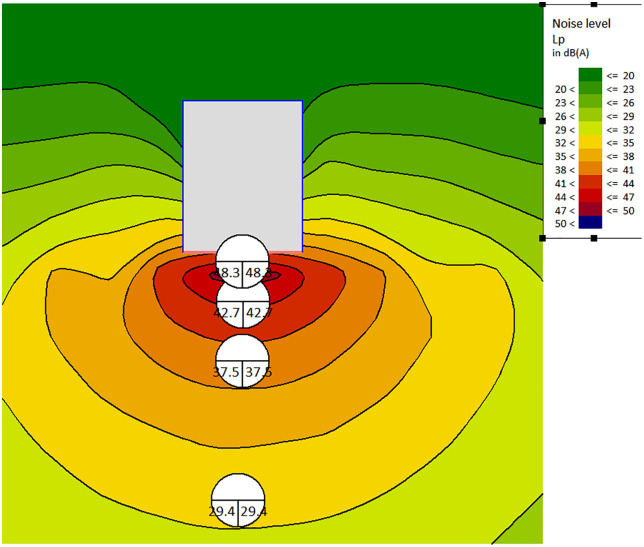
Enclosure wall improvement.

The results demonstrate that by properly configuring the enclosure walls around the machinery, noise levels at sensitive noise control points can be reduced by a significant 30–40 dB(A). The noise distribution map of sensitive control points after the wall renovation is shown in Fig 11. The noise reduction effect of aerated concrete block walls is shown in Table 12. This substantial reduction in noise is indicative of the effectiveness of the enclosure in mitigating noise propagation in the controlled environment.

**Table 12:** The noise attenuation with aerated concrete blocks wall.

Distance (m)	Inside	1	5	10	20
Noise SPL (dBA)	85	48.3	42.7	37.5	29.4

To verify the scalability of the proposed control scheme in diverse environments and compare its time efficiency, low temperature (−25 °C), normal temperature (25 °C), and high temperature (45 °C) environments were selected. The stability and robustness indicators were the noise reduction, noise reduction coefficient of variation, and fluctuation range of each environment. Particle swarm optimization (PSO) algorithm and traditional combination noise reduction method were selected as comparison methods. The experimental results are shown in Table 13.

**Table 13:** Comparison results of noise reduction performance and time complexity of methods in different environments.

Environment Type	Proposed method	PSO	Traditional combination denoising method
Low temperature (−25 °C) noise reduction amount/dB	38.5	33.2	26.1
Noise reduction at room temperature (25 °C)/dB	40.2	36.5	29.3
High temperature (45 °C) noise reduction amount/dB	39.7	34.8	27.5
Coefficient of variation of noise reduction	0.04	0.09	0.16
Noise reduction fluctuation range/dB	1.7	3.3	3.2
Total algorithm time/h	56.3	101.5	63.8

According to Table 13 analysis, the noise reduction achieved by our algorithm in five different environments remained between 38.5-40.2dB, with a coefficient of variation of only 0.04 and a fluctuation range of 1.7dB. This significantly outperforms the PSO algorithm and traditional methods, demonstrating stronger environmental scalability and stability. Its total time consumption is also lower than traditional methods and PSO algorithms, and the noise reduction in each environment is significantly ahead, indicating that the proposed scheme can still maintain efficient and stable noise reduction performance in complex environments, and performs better. The proposed method is based on a genetic algorithm driven multi measure collaborative optimization strategy, and its theoretical time complexity characteristics are significantly different from other methods: the time complexity of the proposed GA based method is dominated by the iterative process of the genetic algorithm, following O (G × N × D) (where G is the number of iterations, N is the population size, and D is the optimization variable dimension). In the actual substation noise optimization scenario, the input scale is relatively controllable, and G, N, and D are all maintained within a moderate range. Even if the model complexity is slightly increased, it can avoid exponential growth in running time. Although PSO has a similar theoretical time complexity form (O (G × N × D)), it requires more iterations (G) to converge to a stable solution in substation noise optimization. This is because PSO relies on particle velocity updates, which can easily get stuck in local optima and require additional iterations to adjust the search direction, resulting in higher effective time complexity in practical applications. The traditional combination denoising method adopts a heuristic trial and error approach, without a system optimization framework, has uncertain time complexity, and tends towards (O (M × K) (M is the number of measures, K is the number of test scenarios). As the number of noise sources or measures increases, M and K show linear growth, and due to the lack of global optimization logic, redundant calculations are generated, resulting in a significant increase in cumulative running time.

## 6. Conclusions

This study conducted separate analyses of the noise characteristics during the construction process and operational phase of a 500kV high-voltage substation. Through the use of SoundPlan simulations, it quantitatively analyzed noise reduction measures applicable to this project. The analysis of noise source characteristics shows that transformer noise is mainly composed of 100 Hz and its harmonics. The sound pressure levels at 100 Hz, 200 Hz, and 400 Hz frequency points are 85dB, 82dB, and 78dB, respectively, accounting for more than 75% of the total noise energy; The fan noise is broadband white noise, with an average sound pressure level of 73dB in the 63–8000 Hz frequency band, and there is a coupling effect with transformer noise. After coupling, the sound pressure level of the noise is increased by 3-5dB compared to when stacked separately. In terms of noise control measures, the optimal height of the sound barrier is 4.5m, which can reduce the noise at the east, south, west, and north boundaries of the substation from 73.4dB, 66.2dB, 47.6dB, 54.6dB to 57dB, 50.7dB, 33.9dB, and 47.8dB, respectively; A 150 mm thick mineral wool board is used in the control room, reducing the noise from 41.2dB to 26.4dB at 1m and from 22.5dB to 8.1dB at 20m, with a maximum noise reduction of 14.8dB. A 200 mm thick B06 aerated concrete block is used to enclose the transformer, which can reduce the internal 85dB noise to 48.3dB at 1m and 29.4dB at 20m, with a noise reduction of 30-40dB. The optimal combination of measures obtained by the optimization algorithm achieves a total noise reduction of 40dB, which is improved by 8–12 dB compared to a single measure optimization. Through Monte Carlo simulation and 30 day on-site testing verification, under the working conditions of transformer load of 70% −130%, ambient temperature of −10–40 °C, and fan speed of 80% −120%, the noise reduction coefficient of variation of the noise control system is 0.06, with a fluctuation range of 2.7dB. It has good stability and robustness, and can provide new technical methods and theoretical support for noise control in high-voltage substations in noise sensitive areas.
